# Comment on Limitations of “Assessing Herpes Zoster Vaccine Efficacy in Patients With Diabetes: A Community‐Based Cohort Study”

**DOI:** 10.1002/jmv.70047

**Published:** 2024-11-19

**Authors:** Rachel A. Cohen, Huifeng Yun, Charles Williams

**Affiliations:** ^1^ GSK Rockville Maryland USA; ^2^ GSK Wavre Belgium

Dear Editor,

We read with interest the Kornelius et al. [1] study on herpes zoster (HZ) vaccination in patients with diabetes mellitus (DM) (2006–2023), concluding that HZ vaccination is not effective in DM patients. The authors acknowledge several limitations: retrospective design; selection bias; no adjustment for DM management; comparison of live‐attenuated zoster vaccine (ZVL) and adjuvanted recombinant zoster vaccine (RZV) with different peak usage periods; possible HZ underdiagnosis; and lack of individual follow‐up data. Here we present additional limitations that should be considered when interpreting the results.

A critical limitation regards use of TriNetX electronic health records (EHRs) for vaccination status to estimate HZ vaccine effectiveness (VE). Most (67%–90%) United States (US) HZ vaccinations are administered in pharmacies without prescriptions (therefore no EHR reporting) [2, 3]. The US TriNetX EHR database includes linked claims data for <10% of the database. Without simultaneous EHR/claims data for most patients, substantial exposure misclassification may arise i.e., ‘no vaccination’ group contains vaccinated patients. The vaccinated group may contain a higher proportion vaccinated in clinical versus pharmacy settings, given the data sources, indicative of multiple/more severe health conditions with potentially more healthcare visits, causing residual confounding. Without overall vaccination rates/settings presented, potential misclassification of exposure or outcomes cannot be assessed. EHR data limitations in ascertaining loss to follow‐up may result in missed HZ infections, which could explain the lower incidence observed versus other studies (Figure 1). Results suggest most patients received only the first RZV dose; however, the proportion of series completion was unclear. Other important missing information included specific disease codes for exclusion, mean follow‐up time, whether censoring was applied (e.g., for death, change of healthcare provider), and proportion of DM types.

**Figure 1:**
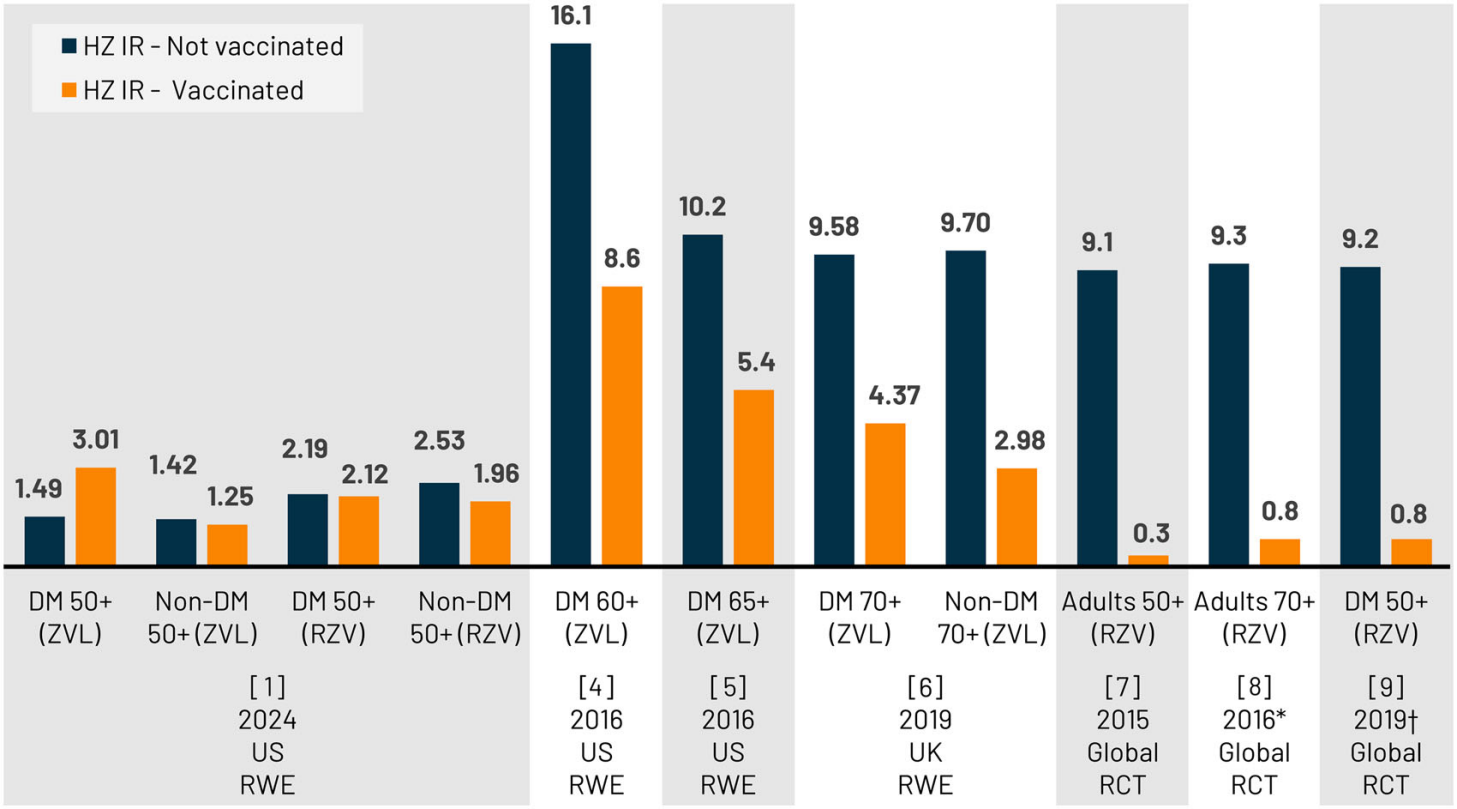
HZ incidence rate (per 1000 person‐years) from RCTs and RWE studies. *Pooled analysis of ZOE‐50/ZOE‐70 [7, 8] study participants; †Post hoc analysis of ZOE‐50/ZOE‐70 participants [7, 8] with ≥1 of 15 medical conditions at enrollment; Abbreviation: DM, diabetes mellitus; HZ, herpes zoster; IR, incidence rate; RCT, randomized controlled trial; RWE, real‐world evidence; RZV, adjuvanted recombinant zoster vaccine; UK, United Kingdom; US, United States; ZVL, live‐attenuated zoster vaccine.

The study assigned different index dates for vaccinated versus unvaccinated (first recorded vaccination date vs. initial DM diagnosis date), which likely led to immortal time bias. By design, the vaccination date always followed the DM date, so only vaccinated patients had a period between diagnosis and vaccination where they were excluded for HZ events. This means that they would need to be followed for longer to experience the exposure, systematically shortening the period for HZ events versus unvaccinated patients, which could substantially bias incidence rates (IRs) in both groups.

The authors stated they lacked individual follow‐up data in their dataset. They did not use person‐time correctly to estimate IR, but instead used the maximum possible follow‐up period (12 years for ZVL; 7 years for RZV). Kaplan–Meier method to estimate both IRs and hazard ratios (HRs) requires individual time‐to‐event data [4]. If IRs estimated by Kaplan–Meier method were used to calculate HRs via Cox proportional hazard regression, this may have led to inaccurate estimates of HZ incidence and HR. Indeed, reported HZ IRs are surprisingly low in the unvaccinated groups versus randomized controlled trials (RCTs) and real‐world evidence (RWE) studies [4, 5, 6, 7, 8, 9]. The ZVL DM cohort IR was higher in vaccinated versus unvaccinated individuals (3.01 vs. 1.49/1000 person‐years), highlighting inconsistencies in results versus the current body of evidence [4, 5, 6, 7, 8, 9].

The DM HZ‐vaccinated population may not be representative of the general DM population, as over 50% did not receive DM treatment. Before matching, vaccinated cohorts had more females, comorbidities, and DM medication use, indicating more severe disease/higher baseline HZ risk versus unvaccinated cohorts. Even after propensity score matching, imbalances remained (e.g., proportion with chronic kidney disease, dementia, and insulin use), which may have affected results.

The authors proposed that discrepancies between their results and other studies may be due to study design and population differences, reduced immunity/responses to vaccines in DM patients, and potential pre‐existing varicella‐zoster virus (VZV) immunity to explain the small differences found between vaccinated and unvaccinated groups. This is counterintuitive, given HZ vaccines are indicated/used in individuals with pre‐existing immunity to VZV [10, 11] and multiple RCTs (including DM patients) and RWE studies demonstrated significant benefits with vaccination (Figure 2) [7, 8, 9, 12, 13, 14, 15, 16, 17]. Furthermore, in six RCTs in immunocompromised populations, RZV demonstrated a robust immune response, and a vaccine efficacy of 68%–87% (in three analyses of RCTs) [18].

**Figure 2:**
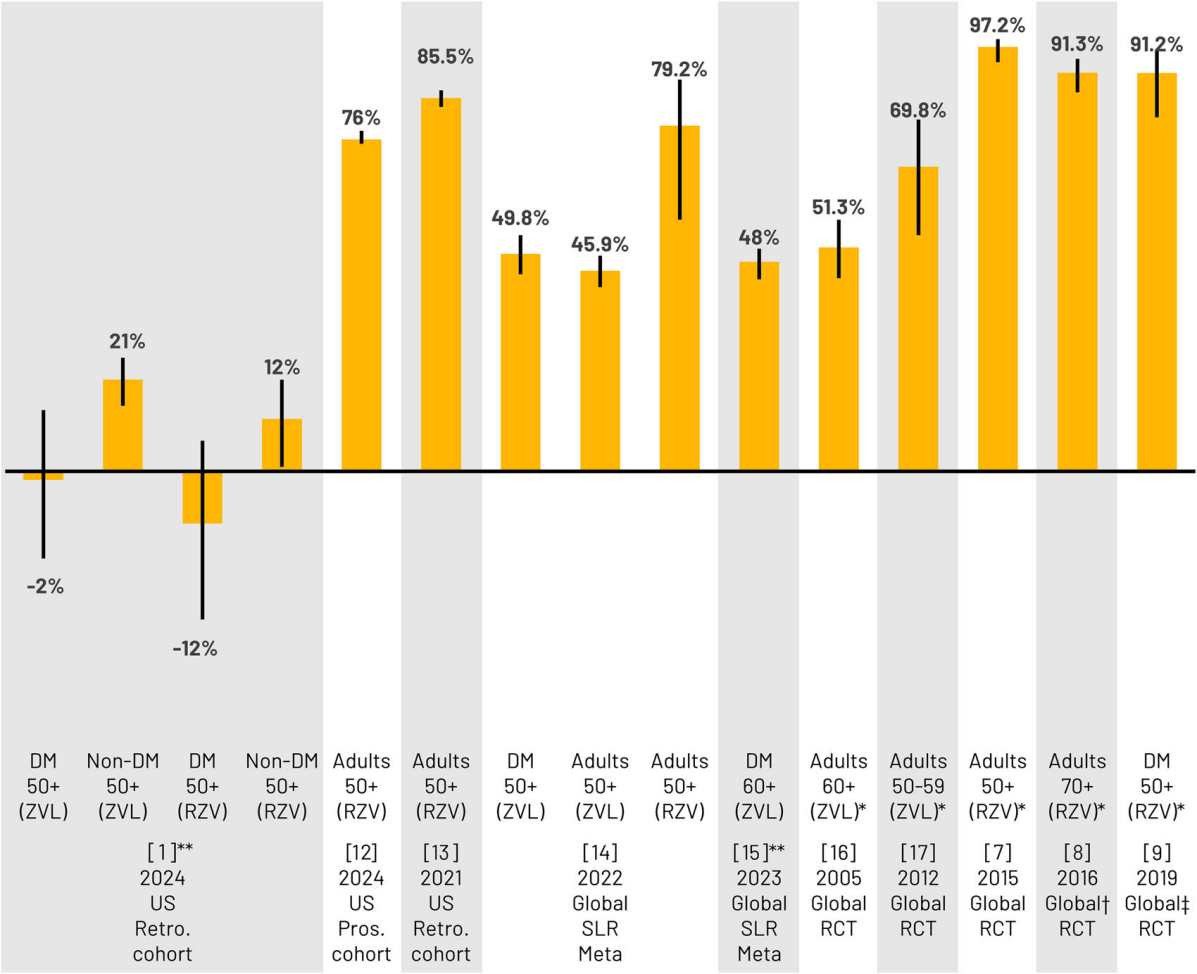
HZ vaccine efficacy*/effectiveness (% and 95% CI) in adults ≥50 years from RCTs and RWE studies. **VE calculated from HR: VE (%) = [1 − HR] × 100 †Pooled analysis of ZOE‐50/ZOE‐70 [7, 8] study participants; ‡Post hoc analysis of ZOE‐50/ZOE‐70 [7, 8] participants with ≥1 of 15 medical conditions at enrollment; CI, confidence interval; DM, diabetes mellitus; HZ, herpes zoster; Pros, prospective; Retro, retrospective; RCT, randomized controlled trial; RWE, real‐world evidence; RZV, adjuvanted recombinant zoster vaccine; SLR Meta, systematic literature review, meta‐analysis; US, United States; ZVL, live‐attenuated zoster vaccine.

In summary, the study results appear questionable given the unsuitable use of the database, missing information, study design and methodologic issues, imbalance in baseline demographics, and inconsistencies with current evidence so the estimates may not represent the true association due to bias. While we appreciate that this study looks to add to the existing literature, it remains critical to evaluate dataset limitations for specific analyses, to determine if the exposure and outcome can be ascertained with adequate validity, and whether sufficient information is present to allow valid estimates. Analyses in claims datasets or combined EHR/claims data would likely generate more accurate VE estimates.

## Author Contributions

All authors participated in the analysis and interpretation of the study; and the development of this Letter. All authors had full access to the data and gave final approval before submission. All authors agree to be accountable for all aspects of the work.

## Ethics Statement

The authors have nothing to report.

## Consent

The authors have nothing to report.

## Conflicts of Interest

Rachel A. Cohen, Huifeng Yun, and Charles Williams are employees of GSK. Rachel A. Cohen and Huifeng Yun hold financial equities in GSK.

## Data Availability

Data sharing is not applicable to this article as no datasets were generated or analyzed during the current study.
